# Immunogenicity profile in African green monkeys of a vaccine candidate based on a mutated form of human Interleukin-15

**DOI:** 10.1186/s12865-021-00470-4

**Published:** 2021-12-18

**Authors:** Yunier Rodríguez-Álvarez, Lino Gerardo Batista-Roche, Alexey Llopiz-Arzuaga, Pedro Puente-Pérez, Rafael Martínez-Castillo, Jorge Castro-Velazco, Alicia Santos-Savio

**Affiliations:** 1 Pharmaceutical Department, Center for Genetic Engineering and Biotechnology, Avenue 31, PO Box 6162, 10 600 Havana, Cuba; 2 Chemistry and Physics Department, Center for Genetic Engineering and Biotechnology, Avenue 31, PO Box 6162, 10 600 Havana, Cuba; 3 Animal Facility Department, Center for Genetic Engineering and Biotechnology, Avenue 31, PO Box 6162, 10 600 Havana, Cuba

**Keywords:** IL-15, IL-15 D8SQ108S, Vaccine candidate, Neutralizing antibodies, African green monkeys, CTLL-2 cells

## Abstract

**Background:**

Interleukin (IL)-15 is a proinflammatory T-cell growth factor overexpressed in several autoimmune diseases such as rheumatoid arthritis. Our initial strategy to neutralize the increased levels of IL-15 consisted in a vaccine candidate based on the recombinant modified human IL-15 (mhIL-15) mixed with the alum adjuvant. A previous study in non-human primates *Macaca fascicularis* has shown that vaccination induces neutralizing antibodies against native IL-15, without affecting animal behavior, clinical status, or the percentage of IL-15-dependent cell populations. However, the mhIL-15 used as an antigen was active in the IL-2-dependent cytotoxic T-cell line CTLL-2, which could hinder its therapeutic application. The current article evaluated the immunogenicity in African green monkeys of a vaccine candidate based on IL-15 mutant D8SQ108S, an inactive form of human IL-15.

**Results:**

IL-15 D8SQ108S was inactive in the CTLL-2 bioassay but was able to competitively inhibit the biological activity of human IL-15. Immunization with 200 µg of IL-15 mutant combined with alum elicited anti-IL-15 IgG antibodies after the second and third immunizations. The median values of anti-IL-15 antibody titers were slightly higher than those generated in animals immunized with 200 µg of mhIL-15. The highest antibody titers were induced after the third immunization in monkeys vaccinated with 350 µg of IL-15 D8SQ108S. In addition, sera from immunized animals inhibited the biological activity of human IL-15 in CTLL-2 cells. The maximum neutralizing effect was observed after the third immunization in sera of monkeys vaccinated with the highest dose of the IL-15 mutant. These sera also inhibited the proliferative activity of simian IL-15 in the CTLL-2 bioassay and did not affect the IL-2-induced proliferation of the aforementioned T-cell line. Finally, it was observed that vaccination neither affects the animal behavior nor the general clinical parameters of immunized monkeys.

**Conclusion:**

Immunization with inactive IL-15 D8SQ108S mixed with alum generated neutralizing antibodies specific for human IL-15 in African green monkeys. Based on this fact, the current vaccine candidate could be more effective than the one based on biologically active mhIL-15 for treating autoimmune disorders involving an uncontrolled overproduction of IL-15.

## Background

Interleukin (IL)-15 was discovered in 1994 by its ability to stimulate the proliferation of the IL-2-dependent T-cell line CTLL-2 [1, 2]. This cytokine plays an essential role in the function and homeostasis of natural killer (NK) cells and T-cell populations [3–5]. IL-15 utilizes the β- and γ-subunits of the IL-2 receptor (IL-2/IL-15Rβγc) and its high-affinity private α subunit IL-15Rα for intracellular signaling in target cells [6, 7]. Despite the widespread expression of the IL-15 messenger RNA in numerous cell types and tissues, the protein expression is controlled at transcription, translation, and intracellular trafficking levels [8, 9]. However, IL-15 overexpression has been associated with the pathogenesis and development of several autoimmune diseases, including rheumatoid arthritis (RA), ulcerative colitis, systemic lupus erythematosus and multiple sclerosis [10–13].

Currently, more than 20 anti-cytokine vaccination approaches for treating the autoimmune disorders mentioned above are in pre-clinical evaluation and clinical trials [14]. These vaccine candidates are composed either of modified entire cytokine or their related peptides linked to various carrier proteins. In particular, chemically-inactivated human tumour necrosis factor (TNF)-α coupled to keyhole limpet hemocyanin (KLH) was assessed extensively in animal models of arthritis [15–17] and clinical trials [18]. Another promising anti-cytokine vaccination approach for treating RA is based on the recombinant modified human IL-15 (mhIL-15) as an antigen, combined with the aluminum hydroxide (alum) adjuvant [19]. Previous experimental studies have shown that active immunization with the vaccine candidate elicits neutralizing antibodies against human IL-15 (huIL-15) in non-human primates (NHP) *Macaca fascicularis*. In addition, vaccination with 200 µg of mhIL-15 did not affect animal behavior, clinical status, blood biochemistry or the percentage of IL-15-dependent cell populations [19]. Nevertheless, the mhIL-15 used as an antigen was active in the CTLL-2 cell proliferation assay [20], which could hinder its therapeutic application considering that IL-15 has been recognized as a T-cell growth factor [5]. Several observations support the key role of T cells in the pathogenesis of autoimmune disorders associated with elevated IL-15 expression [21–24].

At the end of the 1990s, Pettit et al. [25] described two mutants of huIL-15, IL-15D8S and IL-15Q108S, with substitutions in IL-15 sites which are important for binding to the β- and γ-subunits of the IL-15 receptor (mutant D8S: aspartic acid at position 8 was substituted for serine, a mutation in the β-chain interaction site; mutant Q108S: glutamine at position 108 was replaced by serine, a mutation in the γ-chain interaction site). Both mutant proteins block the binding of IL-15 to the β and γ receptor subunits and signal transduction and, consequently, were inactive in the CTLL-2 bioassay. Subsequently, the IL-15 mutant D8SQ108S (hereafter denominated IL-15 D8SQ108S) was used to examine the binding epitope of human monoclonal antibodies against IL-15 [26]. Although the mutant D8SQ108S has already been tested in enzyme-linked immunosorbent assay (ELISA) [26], it has never been used as an antigen for active vaccination. Therefore, this work was aimed to assess the immunogenicity in African green monkeys (AGM) of the vaccine candidate based on IL-15 D8SQ108S, as a potential strategy for treating autoimmune disorders involving IL-15 overexpression. Previously, IL-15 D8SQ108S was expressed in *Escherichia coli* and purified following the same procedure previously described for obtaining the recombinant simian IL-15 (siIL-15) [27]. In this article, the biological activity of the purified protein was determined in the CTLL-2 cell proliferation assay. In order to evaluate the immunogenicity of IL-15 D8SQ108S, healthy AGM were vaccinated with the 200 µg and 350 µg antigen doses combined with the alum adjuvant. Furthermore, a group of animals vaccinated with 200 µg of mhIL-15 mixed with the adjuvant was included in the immunization scheme. During the study, the effects of vaccination on the general clinical parameters and animal behavior of immunized monkeys were examined. The antibody response was analyzed by serum antigen-specific antibody titers. The recognition of huIL-15 and siIL-15 by sera from vaccinated monkeys was also assessed using an ELISA assay. Additionally, the neutralizing capacity of the resulting sera was determined in CTLL-2 cells stimulated with huIL-15 and siIL-15. Lastly, the effect of immune sera on the IL-2-induced proliferation of CTLL-2 cells was studied.

## Results

### Purification and characterization of IL-15 D8SQ108S

**Fig. 1:**
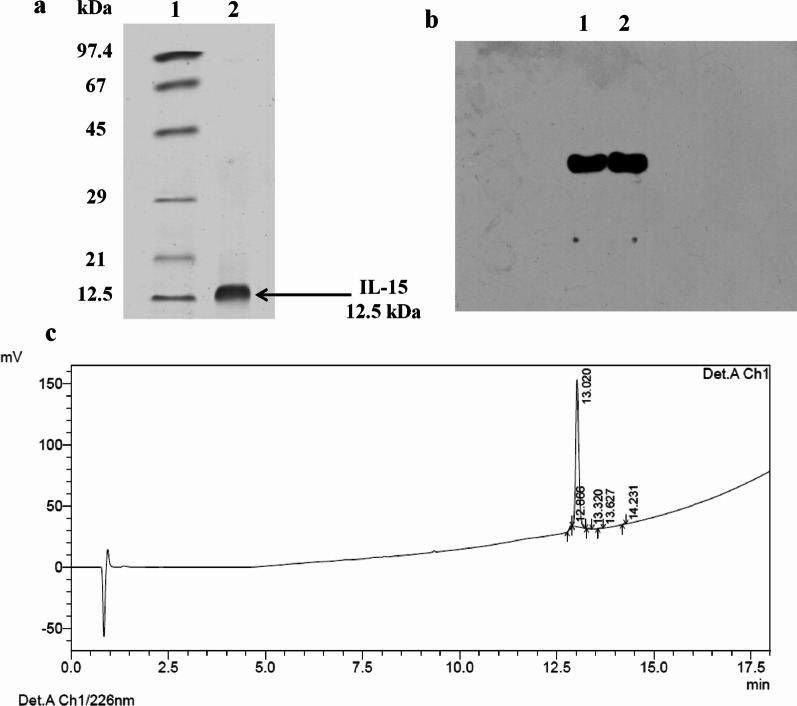
Characterization of purified IL-15 D8SQ108S. **a** SDS-PAGE and **b** Western blot analysis of purified IL-15 D8SQ108S. Five hundred microliters of the main peak collected from the RP chromatography were concentrated, then 5 µg of purified IL-15 D8SQ108S (lane 2) were loaded onto a 15% SDS-PAGE gel. Lane 1: protein molecular weight marker (12.5–97.4 kDa). The anti-huIL-15 monoclonal antibody MAB 9 was used to detect the protein of interest. Lane 1: 5 µg of purified IL-15 D8SQ108S. Lane 2: 5 µg of commercial huIL-15 (positive control). **c** Determination of IL-15 D8SQ108S purity by RP-HPLC. Fifty micrograms of the purified protein were injected into a C_8_ column, obtaining a principal peak with 98% purity

The final preparation of IL-15 D8SQ108S was analyzed by sodium dodecyl sulfate polyacrylamide gel electrophoresis (SDS-PAGE). Figure 1a depicts a major band at 12.5 kDa, corresponding to the expected size for non-glycosylated IL-15. The identity of the purified protein was verified by Western-blot analysis, revealing that IL-15 D8SQ108S was the main protein in the preparation (Fig. 1b). The reverse phase (RP)-high-performance liquid chromatography (HPLC) analysis shows a peak at 13.02 min, which corresponds to the retention time of the mutated form of huIL-15, and a purity of 98% (Fig. 1c).

### Biological activity of IL-15 D8SQ108S in CTLL-2 cells

**Fig. 2:**
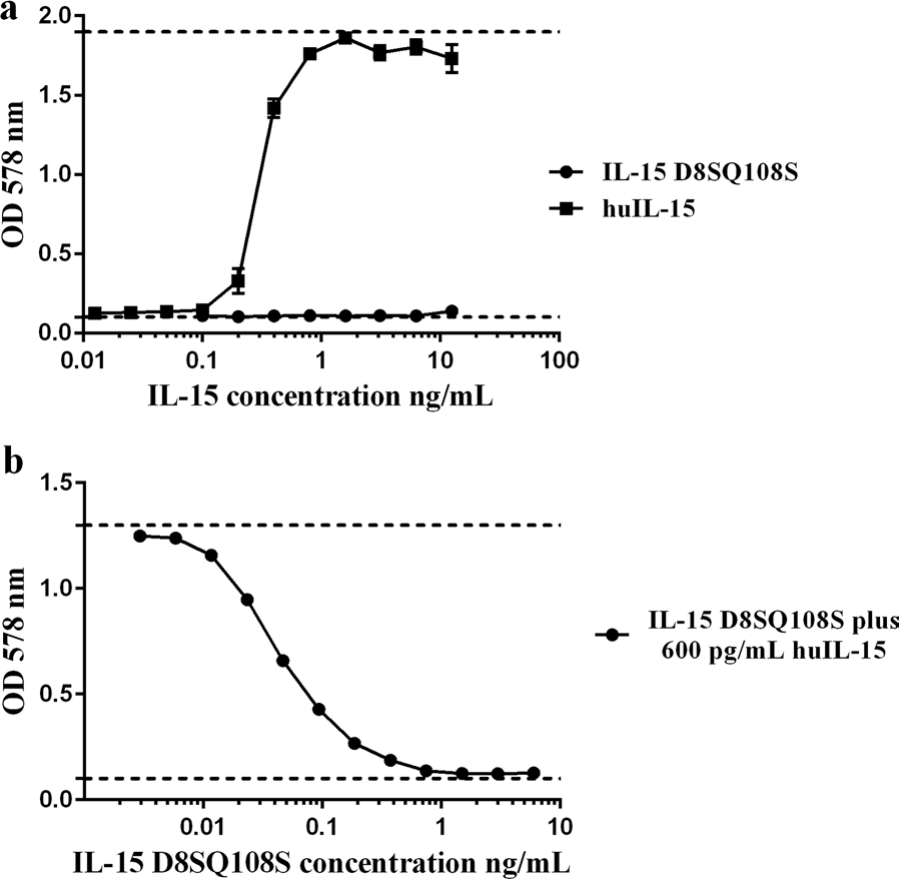
Effect of IL-15 D8SQ108S on the proliferation of CTLL-2 cells. **a** CTLL-2 cells (5 × 10^3^ cells/well) were cultured for 72 h with serial dilutions 1:2 of IL-15 D8SQ108S or commercial huIL-15 with a starting concentration of 12 ng/mL, **b** CTLL-2 cells were cultured with 600 pg/mL of huIL-15 plus serial dilutions 1:2 of IL-15 D8SQ108S (initial concentration 6 ng/mL). Cell proliferation was measured by MTT staining. Data represent the mean values of OD_578nm_ ± standard deviation from two independent experiments performed in triplicate. Dashed lines represent the minimum and maximum proliferation of the cells

The biological activity of the purified protein was measured using the CTLL-2 cell proliferation assay. As shown in Fig. 2a, IL-15 D8SQ108S did not induce the proliferation of CTLL-2 cells, while the commercial huIL-15, used as a positive control, was capable in a dose-dependent manner.

Also, competitive proliferation assays were performed to test the effect of the mutated IL-15 on huIL-15-induced cell proliferation. Figure 2b shows that huIL-15-induced CTLL-2 cell proliferation decreased in the presence of IL-15 mutant, suggesting that IL-15 D8SQ108S could displace the binding of huIL-15 to IL-15 receptor.

### Antibody response in monkeys immunized with IL-15 D8SQ108S

**Fig. 3:**
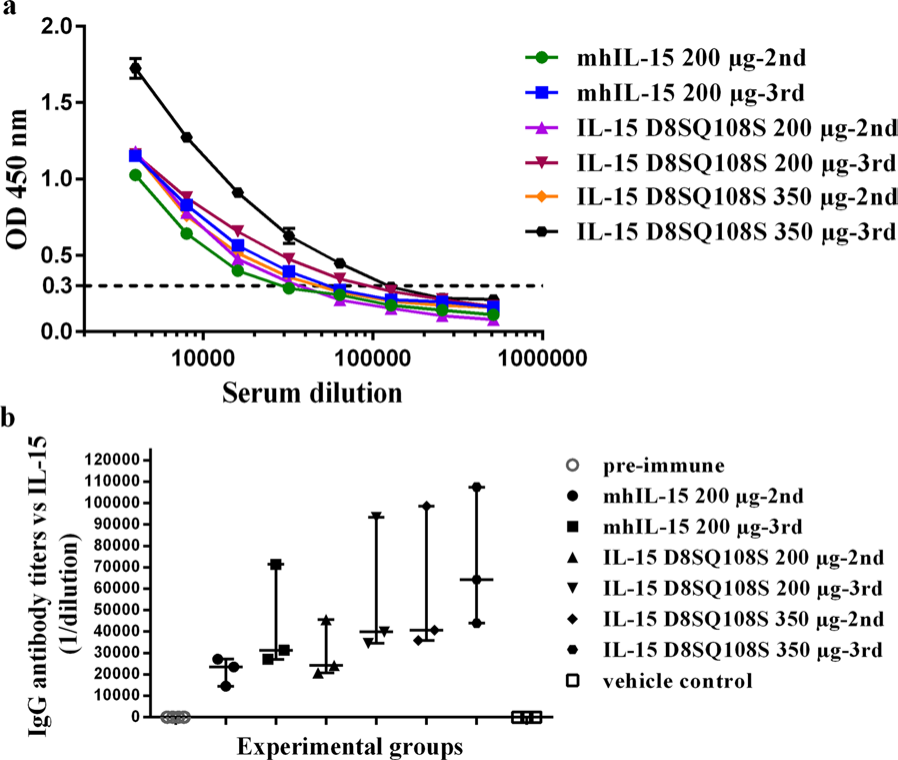
IL-15-specific antibody response elicited in monkeys immunized with IL-15 D8SQ108S or mhIL-15. The ELISA plate was coated with 1 µg/ml of IL-15 D8SQ108S or mhIL-15 and the serum from each animal was evaluated in twofold serial dilutions (starting dilution 1:4000). All sera tested were collected before beginning the scheme (pre-immune) and 15 days after the second and third immunizations. **a** Serum dilution curves of a representative animal per experimental group. Data represent the mean values of OD_450nm_ ± standard deviation (SD) from two independent experiments performed in triplicate. Dashed line represents the value interpolated in the serum dilution curves, which corresponds twice the mean value of pre-immune serum OD_450nm_ from the representative animal of each group. **b** IgG antibody titers against IL-15 in immunized monkeys. The individual antibody titers, expressed as 1/dilution, are represented. The lines represent the median values with interquartile range of antibody titers calculated from duplicate samples of individual monkeys (n = 3) corresponding to each experimental group (200 µg mhIL-15, 200 µg IL-15 D8SQ108S and 350 µg IL-15 D8SQ108S)

In order to evaluate the immunogenicity of IL-15 D8SQ108S, AGM were immunized with 200 µg or 350 µg of antigen combined with the alum adjuvant. In addition, three animals were vaccinated with 200 µg of mhIL-15 in the same adjuvant aiming to compare the immunogenicity of IL-15 D8SQ108S with respect to the non-mutated IL-15. Figure 3 depicts the anti-IL-15 antibody response after the second and third immunizations. A dose–response curve was obtained in serum samples from a representative animal per group (Fig. 3a), which allowed the determination of anti-IL-15 antibody titers. Similar patterns were obtained when the sera of other animals from the same group were assessed. The median values of specific antibody titers were numerically higher than 1:20,000 in all groups, except in the pre-immune and vehicle control monkeys (Fig. 3b). In the 200 µg antigen dose groups, the animals immunized with IL-15 D8SQ108S exhibited higher median values of IgG antibody titers than those receiving mhIL-15 after the second and third immunizations. The highest antibody response was obtained in monkeys vaccinated with 350 µg of IL-15 D8SQ108S, eliciting a median value of specific titer of 1:64,311 after the third immunization.

**Fig. 4:**
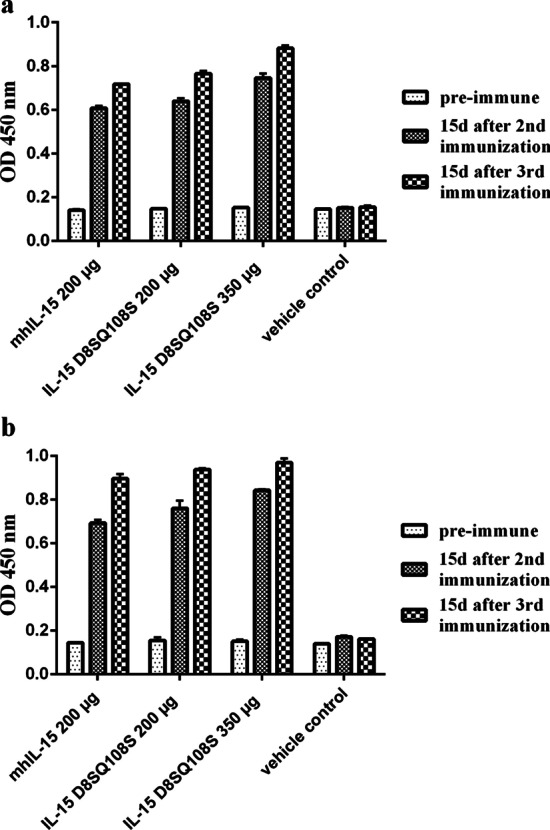
Recognition of huIL-15 (**a**) and siIL-15 (**b**) by sera from immunized monkeys. The ELISA plate was coated with 1 µg/ml of commercial huIL-15 or siIL-15, and it was incubated with the pool of sera from each group (200 µg mhIL-15, 200 µg IL-15 D8SQ108S, 350 µg IL-15 D8SQ108S and vehicle control) diluted 1:4000. All sera tested were collected before beginning the scheme (pre-immune) and 15 days after the second and third immunizations. Columns represent the mean values of OD_450nm_ ± standard deviation (SD) from two independent experiments performed in triplicate

Moreover, the capacity of sera from AGM vaccinated with IL-15 D8SQ108S to recognize the commercial huIL-15 and siIL-15 was determined by ELISA assays. As shown in Fig. 4, the pool of sera per group of immunized animals recognized huIL-15 and siIL-15 immobilized on ELISA plates. Optical density (OD) values in samples of the vaccinated animals were, at least, four-fold those obtained from the vehicle control and pre-immune groups.

### Effect of immune sera on huIL-15 and siIL-15-mediated CTLL-2 cell proliferation

**Fig. 5:**
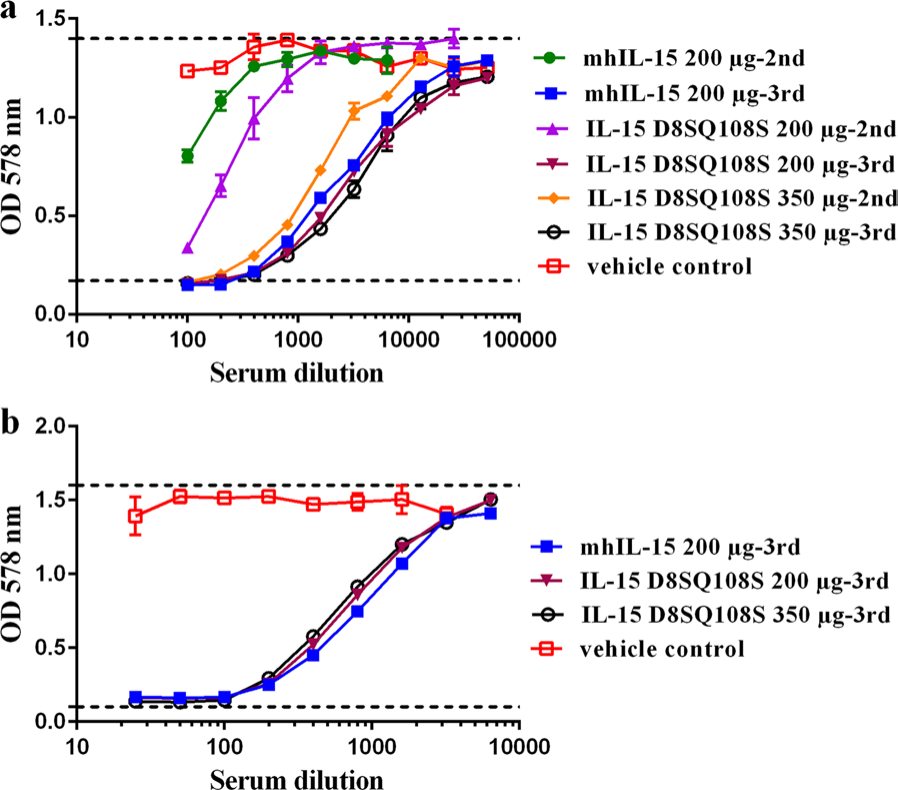
Effect of immune sera on the CTLL-2 cells proliferation induced by huIL-15 and siIL-15. **a** CTLL-2 cells (5 × 10^3^ cells/well) were cultured for 72 h with 300 pg/mL of huIL-15 and serial dilutions 1:2 (initial dilution 1:100) of the pool of sera collected 15 days after the second and third immunizations from animals immunized with 200 µg of mhIL-15, 200 µg of IL-15 D8SQ108S or 350 µg of IL-15 D8SQ108S. **b** CTLL-2 cells (5 × 10^3^ cells/well) were cultured for 72 h with 300 pg/mL of siIL-15 and serial dilutions 1:2 (initial dilution 1:25) of the pool of sera collected 15 days after the third immunization from animals immunized with the same antigen doses mentioned above. Cell proliferation was measured by MTT staining. Data represent the mean values of OD_578nm_ ± standard deviation (SD) from two independent experiments performed in triplicate. Dashed lines represent the minimum and maximum proliferation of the cells

The effect of IL-15 vaccinated monkey sera on huIL-15-induced CTLL-2 cell proliferation was tested in proliferation assays. Table 1 summarizes the neutralizing titers of the pool of sera per group after the second and third immunizations. The cell proliferation curves are depicted in Fig. 5a. Sera of animals immunized with 200 µg of IL-15 D8SQ108S showed a higher neutralizing effect than sera of monkeys vaccinated with the same dose of mhIL-15 (Table 1, Fig. 5a). Additionally, sera collected 15 days after the third immunization showed the highest neutralizing capacity on huIL-15-induced proliferation of CTLL-2 cells, with average titers over 1:2000. Noteworthy, the highest neutralizing effect was observed in sera from animals vaccinated with 350 µg of IL-15 D8SQ108S, with a half-inhibitory dilution (ID_50_) value of 1:3576. In fact, sera of these animals elicited a neutralizing titer of 1:1308 after the first two immunizations (Table 1, Fig. 5a). The commercial neutralizing anti-huIL-15 antibody MAB 2471, used as a positive control, exhibited a dose-dependent inhibition with a neutralization dose of 0.5 µg/mL.

**Table 1:** Serum neutralizing antibody titers of AGM immunized with IL-15D8SQ108S or mhIL-15

Protein variant	Quantity of IL-15/µg	ID_50_ values in CTLL-2 cells	
		2nd dose	3rd dose
mhIL-15	200	1:80	1:2256
IL-15 D8SQ108S	200	1:206	1:2836
IL-15 D8SQ108S	350	1:1308	1:3576

Titers were expressed as the dilution of the pool of sera per group that is required to inhibit the IL-15-induced proliferation of CTLL-2 cells by at least 50%. All sera tested were collected 15 days after the second and third immunizations.

Furthermore, the effect of sera collected 15 days after the third immunization on the CTLL-2 cells proliferation induced by siIL-15 was evaluated (Fig. 5b). Sera from immunized animals inhibited siIL-15-mediated CTLL-2 cell proliferation and showed a neutralizing effect in a dose-dependent manner. The calculated ID_50_ values were 1:750, 1:626 and 1:650 for the group of animals immunized with 200 µg mhIL-15, 200 µg IL-15 D8SQ108S and 350 µg IL-15 D8SQ108S, respectively.

### Effect of immune sera on human IL-2 proliferative activity in CTLL-2 cells

**Fig. 6:**
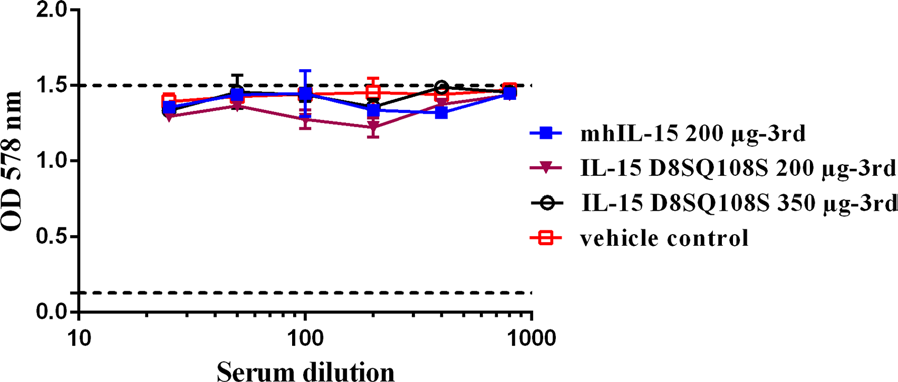
Effect of immune sera on huIL-2-induced proliferation of CTLL-2 cells. CTLL-2 cells (5 × 10^3^ cells/well) were cultured for 72 h with 50 ng/mL of huIL-2 and serial dilutions 1:2 (initial dilution 1:25) of the pool of sera collected 15 days after the third immunization from animals immunized with 200 µg of mhIL-15, 200 µg of IL-15 D8SQ108S or 350 µg of IL-15 D8SQ108S. Cell proliferation was measured by MTT staining. Data represent the mean values of OD_578nm_ ± standard deviation (SD) from two independent experiments performed in triplicate. Dashed lines represent the minimum and maximum proliferation of the cells

The specificity of the neutralizing capacity of sera collected after the third immunization was evaluated using a CTLL-2 cell proliferation assay in the presence of human IL-2 (huIL-2). As shown in Fig. 6, the anti-IL-15 antibodies from pooled sera did not affect the huIL-2-induced proliferation of CTLL-2 cells in any group. Only the commercial neutralizing anti-huIL-2 antibody MAB 202, used as a positive control, exhibited a dose-dependent inhibition with a neutralization dose of 0.5 µg/mL.

### Effects of vaccination on the general clinical parameters and animal behavior of immunized monkeys

During the immunization scheme, no differences were observed in the immunized animals compared to the vehicle control group or the initial clinical observations including body weight, rectal temperature, and respiratory and cardiac rates. Additionally, no changes in animal behavior were observed. All immunized monkeys were apparently healthy during the observational time of the study.

## Discussion

Generally, the target cytokines of anti-cytokine therapeutic vaccines are chemically converted into biologically inactive but still immunogenic derivatives. They are engineered that way to avoid the immune effects that could affect vaccine-induced immune response [28]. Other anti-cytokine strategies employ the cytokine mutated at specific amino acid residues involved in receptor binding and signal transduction [29, 30]. Both approaches are aimed to induce high titers of polyclonal antibodies capable of neutralizing the cytokine-induced pathogenic effects in altered tissues, without interfering with their physiological functions [31].

Nowadays, the immunogenicity of a vaccine comprising an inactive form of the huIL-15 protein has not been evaluated yet. The first active anti-IL-15 antibody therapy was reported by Rodríguez-Álvarez et al.and consisted in the immunization with a modified but active recombinant huIL-15 [19]. In this work, the immunogenicity of an IL-15 D8SQ108S-based vaccine candidate combined with the alum adjuvant was assessed in AGM. The IL-15 mutant was expressed in *E. coli* and further purified to 98% purity. IL-15 D8SQ108S was unable to induce the proliferation of IL-2-dependent T-cell line CTLL-2, which is responsive to IL-15 [32]. Moreover, the mutated form of IL-15 specifically inhibited the huIL-15-triggered CTLL-2 cell proliferation. Similar results were obtained by Pettit et al. [25] with the single mutants IL-15D8S and IL-15Q108S. Both mutants were also inactive in the CTLL-2 bioassay. Altogether, the in vitro results suggest that the disruption of binding to the IL-15 receptor β-subunit or the common γ-chain by the single mutants or IL-15 D8SQ108S would prevent signal transduction and IL-15-dependent CTLL-2 cell proliferation. Once IL-15 binds to its high-affinity IL-15Rα expressed on the cell membrane, it is presented *in trans* to neighboring cells expressing the IL-2/IL-15Rβγc heterodimer. After that, three distinct signaling pathways can be activated mediating cell survival and proliferation [33]. The IL-15D8S and IL-15Q108S mutants, as well as IL-15 D8SQ108S, contain specific mutations at the sites of interaction with the receptor β- and/or γ-chains [25]. These mutant proteins would act as IL-15 receptor antagonists, thus preventing the binding and signaling of IL-15 through its receptor and consequently, inhibiting cell proliferation.

The immunogenicity of IL-15 D8SQ108S was tested in AGM *Chlorocebus aethiops sabaeus*, using the same immunization scheme and the 200 µg antigen dose previously evaluated in NHP *Macaca fascicularis* [19]. Furthermore, an additional IL-15 D8SQ108S dose of 350 µg was tested. By comparison, the median values of IgG specific antibody titers of animals vaccinated with 200 µg of IL-15 mutant were slightly higher than those of monkeys receiving 200 µg of mhIL-15. The anti-IL-15 titers were higher than those described by other authors who evaluated the immunogenicity in NHP of the vaccine candidates based on recombinant modified human vascular endothelial growth factor (huVEGF) [34], and hIS200, a peptide derived from human IL-6 [35]. AGM immunized with 200 µg of P64K-huVEGF_KDR_ exhibited average titers of 1:4000 after the third vaccine administration [34]. Meanwhile, *Macaca fascicularis* monkeys vaccinated with 150 µg of hIS200-KLH reached titers of 1:16,000 after the third immunization [35].

Although the anti-IL-15 vaccine candidate does not comprise the target cytokine fused to a carrier protein, such as P64K or KLH, it is possible that the scrambled disulfide bonds pattern in the mhIL-15 protein may favor the exposure of subdominant or cryptic epitopes that promote an effective antibody response against huIL-15 [19]. Despite the formation of disulfide bonds in the amino acid sequence of IL-15 D8SQ108S has not been established, it could be possible that the mutated protein contains the same structural modification of the mhIL-15, since the mutated form of huIL-15 was expressed in the same host than the non-mutated IL-15. Additional experiments are required to determine the disulfide bonds arrangements in the IL-15 mutant, as well as IgG subclasses of antibodies generated by immunization with the IL-15 D8SQ108S-based vaccine candidate.

According to ELISA results, sera from animals immunized with 200 µg of IL-15 mutant showed higher neutralizing titers than those obtained from monkeys vaccinated with the same dose of mhIL-15. Although for some autoantigens there is no linear relationship between the antigen dose increase and the quality and quantity of the elicited immune response [36, 37], the results of this work demonstrate that the maximum antibody titers against IL-15 were generated in monkeys immunized with the highest dose of IL-15 D8SQ108S. Sera of these animals also exhibited the highest neutralizing titers against huIL-15 in CTLL-2 cells, even after the second immunization. This finding provides the basis to select the dose regimen of the anti-IL-15 vaccine that induces an effective humoral response. Nevertheless, an antigen dose escalation study will be conducted for further characterization of the vaccine-elicited neutralizing anti-IL-15 antibodies in NHP. For this study, at least seven animals per group will be required for performing statistical analysis of data.

The adjuvant and the vaccination scheme are other factors influencing the levels and quality of vaccine-elicited antibodies. Alum adjuvant was used in the immunization experiment, taking into account the previous results wherein it generated the highest neutralizing response in *Macaca fascicularis* monkeys immunized with the anti-IL-15 vaccine [19]. Alum adjuvant fundamentally promotes a humoral immune response [38, 39], although more recent studies demonstrated that it could also favor a Th1 cellular response [40, 41]. Further experiments are needed to demonstrate that vaccination with IL-15 D8SQ108S combined with alum does not induce a specific cellular response against huIL-15. Specifically, in anti-cytokine therapeutic vaccines for autoimmune diseases, an anti-cytokine T cell response is not convenient [42]; due to the role of T cell populations in autoimmune disorders such as RA, inflammatory bowel diseases, systemic lupus erythematosus and multiple sclerosis [21–24].

A long-term immunization scheme was used in the present study and in the previous one wherein the immunogenicity of mhIL-15 was assessed [19]. Spaced immunizations with the longest time interval of 60 days between the second and third vaccine administrations were conducted aiming to achieve the affinity maturation of the anti-IL-15 antibodies. However, a short-term immunization scheme in monkeys could also be evaluated by scaling the antigen dose from 200 to 350 µg and 500 µg/vaccination. In this scheme, at least three immunizations could be carried out with an interval of seven days between the first and second, and 21 days between the second and third vaccine administrations.

The in vitro proliferation bioassays demonstrated that the antibodies generated by immunization with IL-15 D8SQ108S recognize the siIL-15 and inhibit siIL-15-mediated CTLL-2 cell proliferation, because of the high homology between huIL-15 and siIL-15 (97% amino acid sequence identity) [2]. As previously discussed [19], this result supports the use of NHP as a suitable animal model to assess the anti-IL-15 vaccine candidate, including its effectiveness in a model of collagen-induced arthritis in monkeys. The latter constitutes an upcoming objective of the present study. Wild-type murine models are not appropriate for evaluating the anti-IL-15 vaccine candidate because mouse IL-15 shares only 73% amino acid sequence identity with huIL-15 [43]. Indeed, elicited antibodies in mice vaccinated with huIL-15 do not inhibit the biological activity of murine IL-15 [44].

The effect of immune sera on IL-2-induced proliferation of CTLL-2 cells was evaluated taking into account that IL-15 and IL-2 share the β- and γ-chains of the IL-2 receptor [6]. The results demonstrate that sera of monkeys vaccinated with IL-15 D8SQ108S did not inhibit the proliferation of CTLL-2 cells induced by huIL-2, confirming that the neutralizing effect of immune sera is specific for IL-15.

The effects of vaccination on the general clinical parameters and animal behavior of immunized monkeys were assessed as preliminary safety elements of the vaccine candidate. Active immunization with IL-15 D8SQ108S was well tolerated, with no negative effects on the clinical signs and normal behavioral state of the animals. Nevertheless, other studies are needed to further characterize the safety profile of the anti-IL-15 vaccine candidate. For example, the effects of vaccination on the functionality of IL-15-dependent cell populations, such as NK cells and CD8^+^ T lymphocytes, and the hematological and blood biochemical parameters of immunized monkeys.

## Conclusions

The immunization with IL-15 D8SQ108S combined with the alum adjuvant induces specific neutralizing antibodies against huIL-15 in AGM. The highest neutralizing response was induced after the third immunization in monkeys vaccinated with the 350 µg antigen dose. The vaccine-elicited antibodies were capable to recognize the huIL-15 and siIL-15 immobilized on ELISA plates. The anti-IL-15 antibodies also inhibited the biological activity of the aforementioned cytokines in the CTLL-2 bioassay, and did not affect the cell proliferation induced by huIL-2. Preliminary safety data revealed that vaccination with the mutated form of huIL-15 neither affects animal behavior nor the general clinical parameters of immunized monkeys. In summary, the current study characterizes the immunogenicity profile in AGM of a vaccine candidate based on an inactive form of huIL-15, which could have greater therapeutic potential than the active mhIL-15 to be used in the treatment of inflammatory diseases involving IL-15 overexpression.

## Methods

### Reagents and chemicals

Reagents and organic solvents for chromatography were of HPLC grade. Roswell Park Memorial Institute (RPMI) medium 1640, L-glutamine, super signal west pico plus chemiluminescent substrate and 3, 3′, 5, 5′-tetramethylbenzidine substrate solution were purchased from Thermo Fisher Scientific (USA). Phosphate-buffered saline (PBS), tris-buffered saline (TBS), gentamicin sulfate, bovine serum albumin (BSA), skimmed milk, NP-40, goat anti-mouse IgG (Fab specific)-peroxidase antibody, rabbit anti-monkey IgG (whole molecule)-peroxidase antibody, trifluoroacetic acid (TFA), acetonitrile and 3-(4, 5-dimethylthiazol-2-yl)-2, 5-diphenyltetrazolium bromide (MTT) were provided by Sigma-Aldrich (USA). Recombinant huIL-2, recombinant huIL-15, anti-huIL-15 antibody MAB 2471 and anti-huIL-2 antibody MAB 202 were obtained from R&D Systems (USA). Methanol and acetic acid were supplied by Sharlab (Spain). Bromophenol blue and urea were purchased from Merck (USA). Coomassie brilliant blue R-250 was provided by Bio-Rad (USA). Sephadex G-25 Fine was obtained from Pharmacia (UK) and Q Sepharose Fast Flow was supplied by GE Healthcare (USA). Tween 20 was acquired from Calbiochem (Germany). Fetal bovine serum was purchased from Capricorn Scientific (Germany). Ketamine hydrochloride was provided by Liorad Laboratories (Cuba) and alum adjuvant was obtained from Brenntag Biosector (Denmark). Anti-huIL-15 monoclonal antibody MAB 9 was supplied by the Center for Genetic Engineering and Biotechnology (CIGB) of Sancti Spiritu (Cuba).

### Animals

Twelve adult AGM (*Chlorocebus aethiops sabaeus*) of either sex, weighting from 3 to 7 kg, were used. All animals were purchased from the National Center for Animal Breeding (CENPALAB, Havana, Cuba) and maintained in the animal facility at the CIGB (Havana, Cuba). Animals were housed individually in single stainless steel cages (90 × 60 × 60 cm) and randomly distributed into four groups of three animals each to receive IL-15 D8SQ108S or mhIL-15 combined with the alum adjuvant. Three animals were used as vehicle control group. The monkeys were adapted to housing conditions for at least four weeks before starting the immunization protocol. All animals were given ad libitum access to sterile drinking water and fed with standard laboratory food (certificated granulated formula CMQ 1600 ALYco; CENPALAB, Havana, Cuba), according to the species. Monkeys were anesthetized by intramuscular injection of 10 mg/kg ketamine hydrochloride before each vaccine administration. Each animal was observed daily for general health and well-being during the immunization scheme. Body weight, rectal temperature, respiratory and cardiac rates and a general clinical examination were registered before each immunization. The animal experiments were complied by the Guide for the Care and Use of Laboratory Animals recommendations. The experimental protocols were approved by the Animal Care and Usage Committee of the CIGB.

### CTLL-2 cell line

CTLL-2, an IL-2/IL-15-dependent murine T-cell line, was originally acquired from the American Type Culture Collection (TIB-214). The CTLL-2 cell bank was generated by the Biological Assays Laboratories of the CIGB. Cells were grown in RPMI medium 1640 containing 2 mM L-glutamine, 50 µg/mL gentamicin sulfate, 10% heat-inactivated fetal bovine serum and 10 ng/ml recombinant huIL-2. CTLL-2 cells were incubated at 37 °C with 5% CO_2_, 95% humidity and were harvested and used in log phase growth (Cell passage 5 after thawing; cell viability: ≥ 95%). Prior to use, cells were washed five times with RPMI medium.

### Protein purification

IL-15 D8SQ108S, mhIL-15 and siIL-15 proteins were expressed *in E. coli* and purified following the procedure previously described for obtaining siIL-15 [27]. Briefly, the proteins expressed in the insoluble fraction were solubilized in an 8 M urea solution. Then, the insoluble material was eliminated by centrifugation and chaotrope was removed through Sephadex G-25 Fine packed on XK 16/40 column (GE Healthcare Life Sciences, USA). The sample collected from G-25 (without urea) was loaded onto Q Sepharose Fast Flow using a column of 1.6 × 10 cm (GE Healthcare, USA), which was operated at 5 mL/min. After a washing step, IL-15 containing fractions were eluted and the collected sample was applied to a C_4_ column (1 × 25 cm, 10 µm, Vydac, USA) at a flow of 1 mL/min. Proteins were separated using a mobile phase containing solution A (0.1% TFA in water) and solution B (0.1% TFA in acetonitrile), using the same gradient as previously described [27]. Protein separations were monitored at 226 nm. The Bradford method was employed to determine the total protein concentration using BSA as standard [45].

### Reverse phase high-performance liquid chromatography

The RP-HPLC analysis was conducted with 50 µg of purified IL-15 D8SQ108S on Chromolith Performance C_8_ column (4.6 × 100- mm, 2 µm, Merck, USA) with a mobile phase, consisting in solution A (0.1% TFA in water) and solution B (0.1% TFA in acetonitrile) at a flow rate of 2.5 mL/min. A linear gradient was ramped up from 0 to 80% of solution B in 15 min at the same flow mentioned above. The detection wavelength was set at 226 nm.

### Protein electrophoresis and western blot analysis

SDS-PAGE was carried out in a 15% polyacrylamide gel according to the procedure described by Laemmli [46]. The separation was done in Mini-protean chamber (Bio-Rad, USA) at 30 mA until bromophenol blue reach the end of gel. For proteins visualization, the gel was stained with 0.1% Coomassie brilliant blue R-250 for 30 min with gentle shaking. Destaining of gel was accomplished with methanol-acetic acid–water solution (40:10:50, v/v).

For western blot analysis, the proteins on the gel were transferred to a Hybond-C nitrocellulose membrane (Amersham Biosciences, USA) as described by Towbin et al. [47]. The membrane was blocked with 5% skimmed milk in TBS (50 mM Tris–HCl pH 7.6, 150 mM NaCl) at 4 °C during 16 h, washed with TBS, and incubated with 1 µg/mL of anti-huIL-15 monoclonal antibody MAB 9 at 25 °C for 1 h. After washing three times using TBS and TBS-NP (TBS with 0.1% NP-40), the membrane was treated with peroxidase-conjugated goat anti-mouse IgG (Fab specific) antibody at a dilution of 1:1000 for 1 h at 25 °C. Then, the membrane was washed five times and the bands were visualized using a super signal west pico plus chemiluminescent substrate as per the manufacturer’s instructions. All the antibody incubations were performed with 5% skimmed milk in TBS.

### Vaccine doses and schedule

All monkeys were screened for antibodies against IL-15 protein, and considered naive with respect to the antigen when specific antibodies were undetectable by ELISA (titer < 1:50; see methods below). Three groups of monkeys were immunized either with 200 µg of IL-15 D8SQ108S, 350 µg of the same antigen or 200 µg of mhIL-15. The vehicle control group was injected with PBS. All vaccinations were conducted subcutaneously at several sites of the interscapular region, combining the antigen doses or PBS with 1.8 mg/mL of alum, for a total volume of 0.5 mL. Three immunizations were performed, spaced one month between the first and second, and two months between the second and third. Blood samples were collected before beginning the scheme (pre-immune) and 15 days after the second and third immunizations. Complement was inactivated by incubating the sera at 56 °C for 30 min and the sample were then stored at − 20 °C until used. Group serum pools were used in ELISA assays and CTLL-2 cell proliferation bioassays (see methods below). For this purpose, equal volumes of serum from animals of the same group were mixed.

### ELISA for serum anti-IL-15 antibodies

Specific antibody titers against IL-15 were measured through an ELISA as previously described [19]. Briefly, wells were coated with 1 µg of IL-15 D8SQ108S or mhIL-15 in PBS (0.1 M NaCl, 2 mM KCl, 10 mM Na_2_HPO_4_, 1 mM KH_2_PO_4_; pH 7.4) for 16 h at 25 °C. Following blocking step, PBS, 0.05% Tween 20 and 0.01% BSA-diluted sera (twofold serial dilutions from 1:4000 to 1:512,000) were added to wells and incubated for 90 min at 37 °C. After washing step, IgG antibodies were detected with rabbit anti-monkey IgG (whole molecule)-peroxidase antibody diluted 1:10,000 in PBS. After incubating for 1 h at 37 °C, the plates were revealed by incubating with 100 µL of 3, 3′, 5, 5′-tetramethylbenzidine substrate solution. IgG antibody titers were estimated as previously reported [19].

### ELISA for recognition of huIL-15 and siIL-15 by sera from immunized monkeys

To evaluate whether the sera from immunized monkeys recognize the commercial huIL-15 and siIL-15, ELISA assays were performed as previously described by Rodríguez-Álvarez et al. [19, 27]. Briefly, wells were coated with 1 µg/mL of huIL-15 or siIL-15 in PBS for 16 h at 25 °C, and the pool of sera per group, corresponding to 15 days after the second and third immunizations, was evaluated in fixed dilution, 1:4000.

### In vitro CTLL-2 cell proliferation bioassays

The biological activity of IL-15 D8SQ108S was determined through the CTLL-2 cell proliferation assay as previously described [20, 44]. Commercial recombinant huIL-15 was used as a positive control of cell proliferation. In brief, CTLL-2 cells were incubated with two-fold serial dilutions of IL-15 D8SQ108S or commercial huIL-15 (starting concentration 12 ng/mL). After 72 h, cell viability was measured using the MTT assay [48]. The biological activity of the evaluated proteins was measured as their ability to stimulate the proliferation of CTLL-2 cells. To evaluate the effect of IL-15 D8SQ108S on the huIL-15-induced cell proliferation, CTLL-2 cells were cultured with 600 pg/mL of huIL-15 plus serial dilutions 1:2 of IL-15 D8SQ108S (initial concentration 6 ng/mL).

The effect of immune sera on the CTLL-2 cells proliferation induced by huIL-15, siIL-15 and huIL-2 was assessed using the CTLL-2 bioassay as previously described [19, 27, 44]. Briefly, 5 × 10^3^ cells per well were cultured for 72 h with 300 pg/mL of huIL-15 or siIL-15 and serial dilutions 1:2 (starting dilutions 1:100 or 1:25) of the pool of sera per group collected 15 days after the second and third immunizations. MAB 2471, a commercial neutralizing anti-huIL-15 antibody was evaluated in two-fold serial dilutions from 0.03 to 2 µg/mL. Neutralizing titers were expressed as the dilution of sera that is required to inhibit the proliferation of CTLL-2 cells by at least 50%. To assess the effect of immune sera on IL-2 proliferative activity, CTLL-2 cells were incubated with 50 ng/mL of huIL-2 and serial dilutions 1:2 (initial dilution 1:25) of the pool of sera from each group, collected 15 days after the third immunization, or the commercial neutralizing anti-huIL-2 antibody MAB 202 at a concentration range of 0.03–2 µg/mL. Wells containing cells in RPMI medium without cytokine represent the minimum proliferation, while the maximum proliferation corresponds to the cells cultured with 6 ng/mL of IL-15 or 50 ng/mL of IL-2. In all assays, cell proliferation was measured by MTT staining [48].

## Data Availability

All data generated or analyzed during this study are included in this published article in Figs. 1, 2, 3, 4, 5 and 6 and Table 1.

## Abbreviations

AGM: African green monkeys
Alum: Aluminum hydroxide
BSA: Bovine serum albumin
CENPALAB: National Center for Animal Breeding
CIGB: Center for Genetic Engineering and Biotechnology
ELISA: Enzyme-linked immunosorbent assay
HPLC: High-performance liquid chromatography
huIL-2: Human IL-2
huIL-15: Human IL-15
huVEGF: Human vascular endothelial growth factor
ID_50_: Half-inhibitory dilution
IL: Interleukin
IL-15Rα: Interleukin-15 receptor subunit alpha
IL-2/IL-15Rβγc: Beta- and gamma-subunits of the IL-2 receptor
KLH: Keyhole limpet hemocyanin
mhIL-15: Modified human IL-15
MTT: 3-(4, 5-Dimethylthiazol-2-yl)-2, 5-diphenyltetrazolium bromide
NHP: Non-human primates
NK: Natural killer
OD: Optical density
PBS: Phosphate-buffered saline
RA: Rheumatoid arthritis
RP: Reverse phase
RPMI: Roswell Park Memorial Institute
SDS-PAGE: Sodium dodecyl sulfate polyacrylamide gel electrophoresis
siIL-15: Simian IL-15
TBS: Tris-buffered saline
TFA: Trifluoroacetic acid
TNF: Tumour necrosis factor
